# Integration and Visualization of Regulatory Elements and Variations of the *EPAS1* Gene in Human

**DOI:** 10.3390/genes12111793

**Published:** 2021-11-13

**Authors:** Aleša Kristan, Nataša Debeljak, Tanja Kunej

**Affiliations:** 1 Medical Centre for Molecular Biology, Institute of Biochemistry and Molecular Genetics, Faculty of Medicine, University of Ljubljana, 1000 Ljubljana, Slovenia; alesa.kristan@mf.uni-lj.si (A.K.); natasa.debeljak@mf.uni-lj.si (N.D.); 2 Department for Animal Science, Biotechnical Faculty, University of Ljubljana, 1230 Domžale, Slovenia

**Keywords:** *EPAS1* gene, hypoxia-inducible factor (HIF), regulatory elements

## Abstract

Endothelial PAS domain-containing protein 1 (EPAS1), also HIF2α, is an alpha subunit of hypoxia-inducible transcription factor (HIF), which mediates cellular and systemic response to hypoxia. EPAS1 has an important role in the transcription of many hypoxia-responsive genes, however, it has been less researched than HIF1α. The aim of this study was to integrate an increasing number of data on EPAS1 into a map of diverse OMICs elements. Publications, databases, and bioinformatics tools were examined, including Ensembl, MethPrimer, STRING, miRTarBase, COSMIC, and LOVD. The EPAS1 expression, stability, and activity are tightly regulated on several OMICs levels to maintain complex oxygen homeostasis. In the integrative EPAS1 map we included: 31 promoter-binding proteins, 13 interacting miRNAs and one lncRNA, and 16 post-translational modifications regulating EPAS1 protein abundance. EPAS1 has been associated with various cancer types and other diseases. The development of neuroendocrine tumors and erythrocytosis was shown to be associated with 11 somatic and 20 germline variants. The integrative map also includes 12 EPAS1 target genes and 27 interacting proteins. The study introduced the first integrative map of diverse genomics, transcriptomics, proteomics, regulomics, and interactomics data associated with *EPAS1*, to enable a better understanding of EPAS1 activity and regulation and support future research.

## 1. Introduction

Endothelial PAS domain-containing protein 1 (EPAS1), also known as Hypoxia-inducible factor 2 alpha (HIF2α), is an alpha subunit of heterodimeric transcription complex HIF-2. Transcription complexes HIFs function as master regulators of oxygen homeostasis [1]. HIFs regulate numerous responses to hypoxia through transcription activation of multiple genes that either increase oxygen delivery or decrease oxygen consumption. The former group includes gene for erythropoietin (*EPO*), which regulates red blood cell production and gene for vascular endothelial growth factor (*VEGF*), which promotes angiogenesis and increases vascular permeability and local tissue oxygenation. In addition, HIFs regulate genes involved in iron metabolism and bone marrow microenvironment adjustments, which facilitate erythroid progenitor proliferation and maturation. EPAS1 regulation of glycolytic enzymes and glucose transporters decreases oxygen consumption with metabolic switch from oxidative to glycolytic [1,2,3]. Due to hypoxic environment in early developmental stages of embryos, HIFs are also involved in the mammalian embryogenesis [3].

HIF factors are comprised of an alpha subunit, HIFα, and a beta subunit, HIFβ. The first discovered isoform of an alpha subunit was HIF1α [4], while EPAS1 and HIF3α were subsequently identified [5,6,7]. HIF activity depends on the HIFα subunit, whose stability is oxygen-dependent. The transcription factor HIF is inactive at normal oxygen concentration (21% O_2_), as the subunit HIFα is degraded by hydroxylation with the egl-9 family hypoxia inducible factors (EGLN1, 2, and 3), referred also as prolyl hydroxylases PHD1, 2, and 3, and subsequent ubiquitination with the von Hippel-Lindau tumor suppressor protein (VHL). Hydroxylation is an oxygen-dependent post-translational modification, therefore under hypoxic conditions (~1% O_2_), hydroxylase activity is inhibited. A stable HIFα subunit accumulates and forms HIF dimer complex, which activates the transcription of target genes in the nucleus [8,9].

Stable HIF dimer complex binds to the hypoxia-response element (HRE) with core-binding motif RCGTG in the regulatory regions of HIF-target genes [10,11]. At first, only around 70 target genes with HRE binding motifs were known [11], but later experiments indicated that HIF transcriptional activation is far more complex with a larger number of target genes, diverse direct and indirect mechanisms of HIF activity, and the existence of multiple HIFα isoforms with distinct targets [10,12]. The isoforms HIF1α and EPAS1 bind to the identical HREs and have multiple common and also unique transcriptional targets, while the role of isoform HIF3α in the transcriptional regulation is under-researched [10]. The precise mechanisms beyond HIF1α and EPAS1 transcriptional selectivity were a matter of substantial research and are still not fully understood, thus highlighting the complex nature of the cellular response to low oxygen. Cell type, severity and duration of hypoxia, culture conditions, and presence of specific co-activators are among the factors that influence the transcriptional output mediated by different HIFα isoforms [13]. In the acute and intermittent hypoxia (i.e., oxygen levels between normal and hypoxic), more target genes bound HIF1α rather than EPAS1, while after longer periods of hypoxia (>24 h), EPAS1 begins to exert more of an influence [10]. One of the possible explanations for differences in HIFα transcriptional activity is also a tissue-specific *HIFα* expression pattern. *HIF1α* is expressed ubiquitously in all cells, whereas *EPAS1* is expressed only in certain tissues. There is also growing evidence of cooperation with other transcription activators and repressors for modulation of specific HIF activity [14]. 

Integrative map for *HIF1α* was recently published by Kunej (2021) [15]. In comparison to HIF1α, the transcription factor EPAS1 has been less studied in the past and integrative study has not yet been published. However, EPAS1 has a key function in the cellular response to low oxygen, and there has been a growing emphasis on its role in transcriptional activation, especially in association with cancer. The number of publications related to the EPAS1 regulation and activity has been increasing in the last decade and so does the need to systematically integrate information in one place. To support the future research on the EPAS1 and give a more comprehensive understanding into the complex regulation and role of EPAS1 in oxygen-deprived environment, the aim of this study was to integrate relevant multi-omics data of the *EPAS1* gene from publications and databases. 

## 2. Materials and Methods

Information regarding DNA and RNA sequences was collected from genome browsers Ensembl, release 104 (https://www.ensembl.org/index.html, accessed on 22 October 2021) [16], and National Centre for Biotechnology Information (NCBI) (https://www.ncbi.nlm.nih.gov/, accessed on 22 October 2021) [17]. Data about the size of protein sequence, protein expression and localization was retrieved from the Human Protein Atlas, version 20.1 (https://www.proteinatlas.org/, accessed on 22 October 2021) [18]. Information regarding protein domains, regions, functions, and post-translational modifications (PTMs) was gathered mainly from the literature and from UniProt, release 2021_03 (https://www.uniprot.org/, accessed on 9 November 2021) [19]. Nucleotide sequences of 2000 bps upstream and downstream of *EPAS1* transcription start site (TSS) and of 3′UTR region for further analysis of CpG islands and miRNAs were retrieved from Ensembl, release 104. CpG islands analysis was performed using MethPrimer (https://www.urogene.org/methprimer/, accessed on 22 October 2021) [20]. The parameter settings were as follows: island size > 200, GC percent > 50.0, Obs/Exp > 0.60. Locations of promoter regions were obtained from the University of California Santa Cruz (UCSC) Genome browser (https://genome.ucsc.edu/, accessed on 22 October 2021). Protein interactions were retrieved from the Search Tool for the Retrieval of Interacting Genes/Protein (STRING) (https://string-db.org/, accessed on 22 October 2021) [21] and Reactome (https://reactome.org/, accessed on 22 October 2021) [22]. From the STRING we listed only the EPAS1-protein interactions that were experimentally determined. Experimentally validated miRNA targets were obtained from the miRTarBase, release 9.0 beta (https://mirtarbase.cuhk.edu.cn/, accessed on 25 October 2021) [23]. Scientific papers were used to retrieve information about EPAS1 upstream regulators and downstream targets. The GO enrichment analysis for biological processes of downstream targets was performed with Gene Ontology (GO) database (http://geneontology.org/, accessed on 22 October 2021), using PANTHER classification system [24]. Information regarding involvement of EPAS1 in pathophysiology was gathered from the literature and databases Online Mendelian Inheritance in Man (OMIM) (https://omim.org/, accessed on 22 October 2021) [25] and Simple ClinVar (http://simple-clinvar.broadinstitute.org/, accessed on 22 October 2021) [26]. For *EPAS1* variants associated with disorders, we reviewed the literature, the Catalogue of Somatic Mutations in Cancer (COSMIC) (https://cancer.sanger.ac.uk, accessed on 22 October 2021) [27] and the Leiden Open Variation Database (LOVD) (https://www.lovd.nl/, accessed on 22 October 2021) [28]. The article is structured according to the taxonomy of multi-omics levels proposed by Pirih and Kunej (2018) [29]. All reported genomic locations are given based on GRCh38 genome assembly.

## 3. Results

### 3.1. DNA Sequence–Genomics

**Figure 1:**
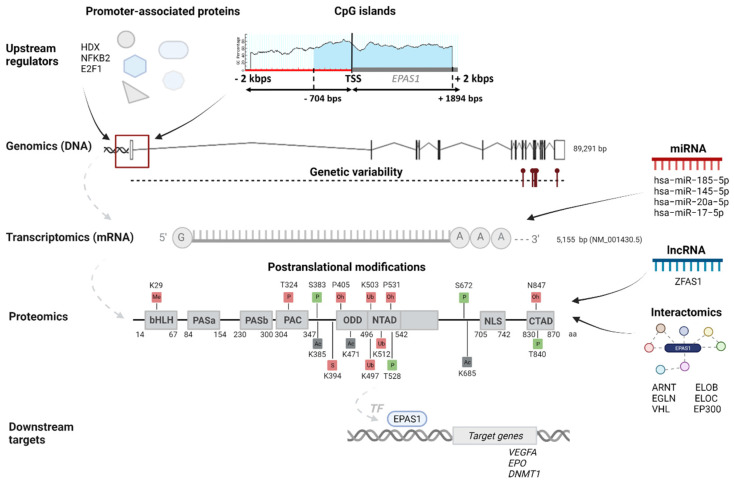
An integrated EPAS1 multi-omics map. An integrative map visualizes important genomics, transcriptomics, proteomics, interactomics data, together with the upstream regulators and downstream targets of the *EPAS1* gene. CpG island is indicated with blue. Post-translational modifications are indicated in squares, with green squares indicating an effect on enhanced EPAS1 stability and activity, red squares indicating an effect on decreased EPAS1 stability and activity, and gray squares indicating ambiguous effect. Me indicates methylation, P indicates phosphorylation, Ac indicates acetylation, S indicates SUMOylation, Ub indicates ubiquitination, and Oh indicates hydroxylation.

*EPAS1* gene has genome size 89291 base pairs (bps) and it is mapped on chromosome 2, cytogenetic band 2p21 (Figure 1) [17]. In the genome browsers Ensembl and NCBI, *EPAS1* is indicated with the ID numbers ENSG00000116016 and 2034, respectively [16,17]. 

### 3.2. RNA Sequence-Transcriptomics

**Figure 2:**
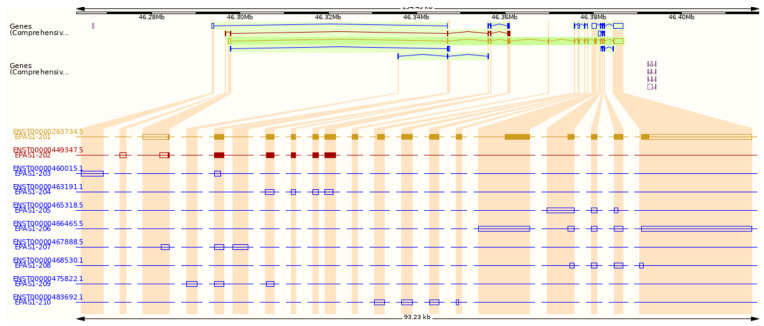
Transcripts of the *EPAS1* gene from the Ensembl genome browser. Gold and red indicate protein-coding transcripts, blue indicates non-coding transcripts.

According to the Ensembl genome browser, the *EPAS1* gene has ten splice variants, two protein-coding, and eight non-coding. (Figure 2). Non-coding transcripts are either processed transcripts without an open reading frame (ORF) or transcripts whereby introns are retained in the mature mRNA. The longest protein-coding transcript is marked with an ID ENST00000263734.5 in Ensembl and NM_001430.5 in NCBI. It comprises 5155 bps and consists of 16 exons (Figure 1 and Figure 2). The shorter protein-coding transcript ENST00000449347.5 is 1067 bps long and has seven exons (Figure 2) [16]. In the NCBI database, another predicted transcript is deposited with an ID XM_011532698.2 [17].

**Figure 3:**
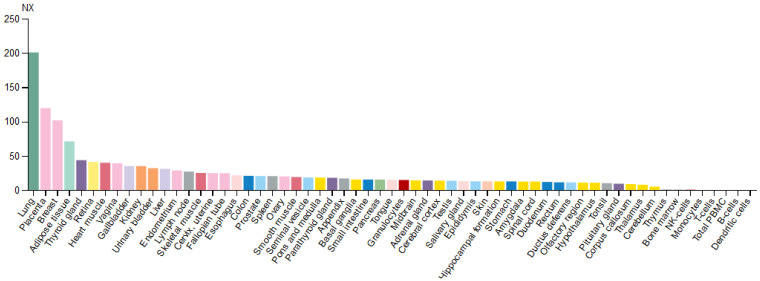
Expression profile of *EPAS1* mRNA in various types of tissues. Normalized expression levels (NX) for 55 tissues and 6 blood cell types are obtained by combining data from the three transcriptomics datasets (HPA, GTEx, and FANTOM5).

Based on the mRNA expression profile obtained from the Human Protein Atlas database, the *EPAS1* gene is expressed in various types of tissues, with enhanced expression in the lungs (Figure 3). In the brain, *EPAS1* mRNA was detected in all regions, while within blood cell lineage it was expressed only in granulocytes (basophils and eosinophils) [18].

### 3.3. Protein Sequence-Proteomics

The longest *EPAS1* transcript NM_001430.5 is coding for the protein sequence marked in NCBI with an ID NP_001421.2. It has 870 amino acids (aa) and a protein mass 96.5 kDa (Figure 1). The shorter transcript ENST00000449347.5 is coding for a protein with 259 aa and mass 29.6 kDa [16,17,18]. The EPAS1 protein is, along with the other two HIFα subunits and the HIFβ subunit, a member of the basic helix-loop-helix Per-ARNT-Sim (bHLH-PAS) family of proteins. It is recognized by strongly conserved bHLH and PAS domains at N-terminus. The bHLH domain is located between 14 and 67 aa and is involved in DNA binding to the HRE in target genes. The region fundamentally required for DNA binding is located from 26–53 aa within the bHLH domain. Two PAS domains, PAS 1 (84–154 aa) and PAS2 (230–300 aa) have a role in the heterodimerization of alpha and beta subunits. The required region for heterodimer formation with ARNT is from 171–192 aa. Next to the PAS domain towards the C-terminus is the PAS-associated C-terminal (PAC) domain (304–347 aa). Important domains for transactivation of HIF target genes are the N-terminal transactivation domain (N-TAD; 496–542 aa) and the C-terminal transactivation domain (C-TAD; 830–870 aa). The N-TAD domain is located inside the highly conserved oxygen-dependent degradation domain (ODDD), where are positioned aa residues responsible for the EGLN1 and VHL binding, controlling stability, and activity of EPAS1 [13,19]. Between TAD domains is the nuclear localization signal (NLS) (705–742 aa), responsible for the translocation of EPAS1 protein into the nucleus (Luo and Shibuya, 2001). Transcript NM_001430.5 has all the above-mentioned domains (Figure 1), while the shorter transcript ENST00000449347.5 (Uniprot number C9J9N2) has only bHLH (14–67 aa) and PAS (92–147 aa) domains [19].

As presented in the Human Protein Atlas and in the literature, the EPAS1 protein is mainly located in the nucleus upon hypoxic induction, but also in the cytosol [18]. Localization in the cytosol is supported by the role of EPAS1 in an oxygen-regulated translation of proteins [30].

### 3.4. Regulomics

Regulomics encompasses all elements that regulate gene expression and EPAS1 protein levels. In this section, we gathered data of *EPAS1* promoter regions and proteins that bind to the promoter and regulate *EPAS1* expression; CpG islands around *EPAS1* promoter regions, which have an important role in the regulation of gene expression due to highly unmethylated sequences; regulatory non-coding RNAs (miRNAs and lncRNAs), which are validated to bind to the *EPAS1* mRNA or protein; and post-translational modifications (PTMs) that regulate EPAS1 protein levels.

#### 3.4.1. Promoter Regions and Promoter-Binding Proteins 

Based on the data from the UCSC genome browser generated by the Eukaryotic Promoter Database, two experimentally validated promoter sequences of 60 bps were identified. The first promoter sequence is located at the genomic loci chr2:46297358-46297417 and starts 49 bps upstream of the TSS. The second identified promoter at the genomic loci chr2:46297717-46297776 is located within the 5′UTR region, starting 310 bps downstream of the TSS.

Regulation of HIFα subunits is mostly considered on the protein level with post-translational modifications. Recently, Hamidian et al. (2018) studied the correlation between high expression of *EPAS1* and neuroblastoma tumor progression and showed that the *EPAS1* gene is in neuroblastoma cells also regulated at the level of DNA transcription. With the novel engineered method, they identified 27 proteins with specific binding to the promoter of *EPAS1* in neuroblastoma cells (Table 1). The majority of identified proteins (24) dissociated in hypoxic conditions, including nucleosome-associated proteins H2ah, H4, and H2bk, which suggests that opening of chromatin surrounding *EPAS1* is an important mechanism for hypoxia-induced *EPAS1* expression. Strong dissociation from the *EPAS1* promoter in hypoxia has also been shown by the highly divergent homeobox (HDX) transcription factor, with a putative role in the negative regulation of *EPAS1* expression [31]. High expression of *EPAS1* was associated with the progression of other types of cancer. Cui et al. (2016) showed that the protein methyl-CpG binding protein 3 (MBD3) binds to the *EPAS1* promoter in breast cancer cells and contributes to enhanced *EPAS1* transcription. MBD3 binds to the CpG-rich promoters and induces demethylation of promoters and active *EPAS1* gene expression [32]. D’Ignazio et al. (2018) found another inducer of the *EPAS1* expression in cancer cells, tumor necrosis factor superfamily member 14 (TNFSF14), also known as LIGHT. LIGHT-induced expression of *EPAS1* requires binding of p52 protein to the *EPAS1* promoter [33]. Transcription factor E2F1 was also proved to regulate *EPAS1* transcription [34] (Table 1).

#### 3.4.2. CpG Islands

A sequence of 4000 bps (±2000 of the TSS) in the *EPAS1* gene was analyzed for the presence of the CpG islands. According to the MethPrimer, one CpG island is densely distributed upstream and downstream of the TSS site (Figure 1). The island length is 2599 bps and is located 704 upstream and 1894 downstream of the TSS. As expected, the identified two promoter regions overlap with the CpG island. It was found by several studies that DNA methylation patterns of CpG island in promoter regions of *EPAS1* gene affected transcriptional activity and progression of several diseases, including breast cancer, colorectal cancer, non-small cell lung cancer (NSCLC), and chronic obstructive pulmonary disease [32,35,36,37,38].

#### 3.4.3. Non-Coding Regulatory RNAs

**Figure 4:**
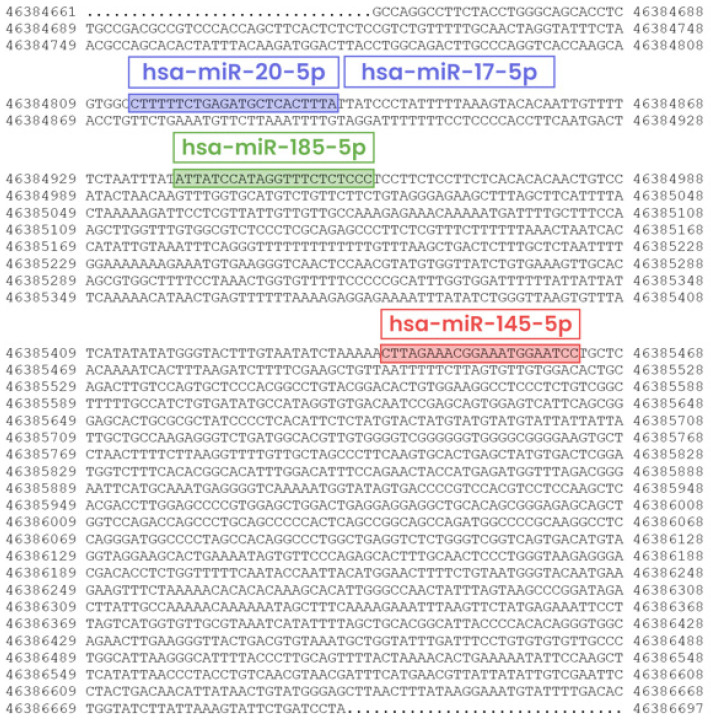
Locations of four miRNAs binding sites on the 3′UTR region of *EPAS1*. Genomic location of the 3′UTR region is from 46,384,661 to 46,386,697 bps (genome assembly GRCh38).

According to the miRTarBase, *EPAS1* mRNA has been experimentally confirmed to be targeted by 13 microRNAs (miRNA) in humans [23]. Among them, four miRNAs hsa-miR-185-5p, hsa-miR-145-5p, hsa-miR-20a-5p, and hsa-miR-17-5p have strong evidence of binding to the *EPAS1* mRNA, which is supported by reporter assays, western blot (WB), or quantitative PCR (qPCR) methods (Table 2). From the publications, we retrieved the target sites of those four miRNAs and marked them on the *EPAS1* 3′UTR region (Figure 4).

The EPAS1 protein has also been a target for long non-coding RNAs (lncRNAs). Zhu et al. (2021) showed that the lncRNA zing finger antisense 1 (ZFAS1) interacted with the EPAS1 protein and up-regulated the protein levels of EPAS1. The exact mechanism for upregulation of EPAS1 is still unknown [39].

#### 3.4.4. Post-Translational Modifications (PTMs)

Under normal oxygen conditions, EPAS1 is continually transcribed and translated; however, normal oxygen tension leads to proteasomal degradation of EPAS1. Proteasomal degradation and regulation of EPAS1 stability and activity involves PTMs (Figure 1). While many of the PTMs occur independently of oxygen tension, the modifications within the ODD domain exist only in normoxia [43]. Prolyl hydroxylase EGLN1 modifies conserved proline residues Pro-405 (P405) and Pro-531 (P531), which all exist within conserved motifs LAPYIXXXDFQL (P is a proline residue targeted as secondary hydroxylation site) and LXXLAP (P is a proline residue targeted as primary hydroxylation site). Proline hydroxylation allows binding of the VHL E3 ubiquitin ligase complex, which poly-ubiquitinates EPAS1 at residues Lys-497 (K497), Lys-503 (K503), or Lys-512 (K512), triggering degradation by the proteasome. Another hydroxylation occurs in the C-TAD domain of EPAS1, on asparagine residue Asn-847 (N847). Enzyme HIF1AN (hypoxia-inducible factor 1 alpha subunit) hydroxylates asparagine residue to interfere with the binding of cofactor CBP/p300 for transcription, thereby diminishing the transactivation potential of EPAS1. Contrarily, phosphorylation of Thr-840 (T840) in the C-TAD domain of EPAS1 has been demonstrated to enhance the transactivation of target genes, by either disrupting interaction with VHL or increasing the affinity of EPAS1 for transcriptional co-activator CBP/p300. Phosphorylation at residues Ser-383 (S383), Thr-528 (T528), and Ser-672 (S672) act on an enhanced transcriptional activity of EPAS1 by regulating nuclear localization and retaining the protein in the nucleus. In addition, phosphorylated residue Thr-324 (T324) is responsible for the downregulation of transcriptional activity by aberrant binding of transcription factor SP1 [13,43]. Acetylation appears to regulate HIFα subunit stability and activity, both positively and negatively. EPAS1 can be acetylated at lysine residues Lys-385 (K385), Lys-685 (K685), and Lys-471 (K471), but there is contradictory evidence concerning the role of this modification [13]. It was reported that sirtuin Sirt1 forms a complex with EPAS1 and through deacetylation of lysine residues enhances its transcriptional activity [44]. Other PTMs could also affect the EPAS1 protein stability and activity. For instance, methylation of Lys-29 (K29) in the bHLH domain induces transcriptional inhibition independent of hydroxylase action, while linkage of small ubiquitin-related modifier (SUMO) at residue Lys-394 (K394) facilitates VHL proteasomal degradation of EPAS1 [43]. Evidence of proteasomal degradation in normoxia is confirmed by low protein expression across different tissue in the Human Protein Atlas [18].

### 3.5. Interactomics

**Figure 5:**
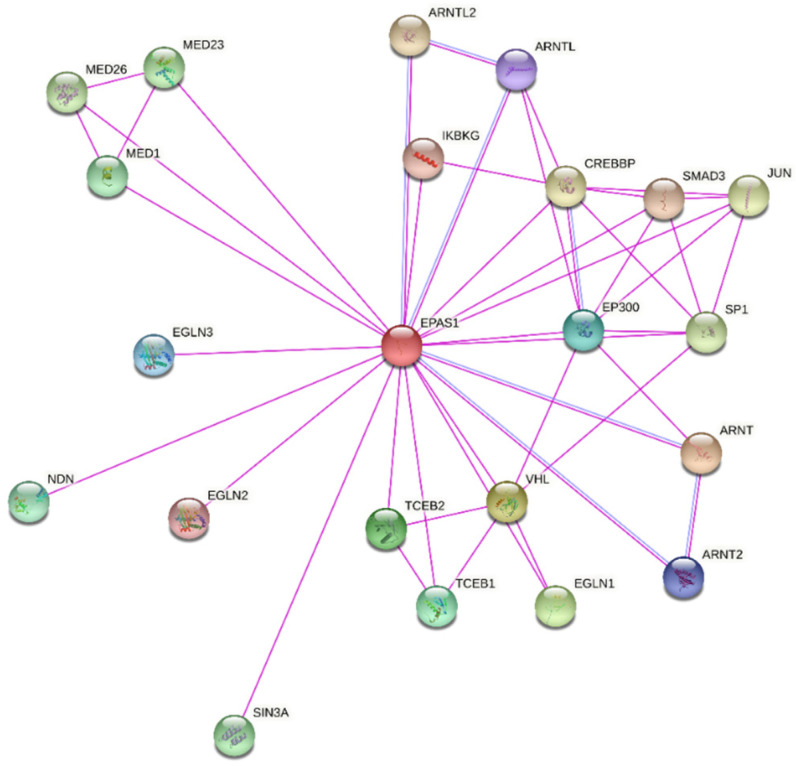
Experimentally determined direct interacting partners of the EPAS1 protein, visualized using STRING tool. Pink lines indicate experimentally determined interactions. Blue lines indicate interactions from curated databases.

According to the STRING database, the EPAS1 protein is directly interacting with 21 proteins, which were experimentally determined (Figure 5). Among the proteins with the strong confidence of interaction (interaction score ≥ 0.700) are Aryl hydrocarbon receptor nuclear translocators (ARNT and ARNT2), Aryl hydrocarbon receptor nuclear translocator-like protein 1 (ARNTL), VHL, EGLN1, EGLN2, EGLN3, Elongin B (ELOB, previous name TCEB2), Elongin C (ELOC, previous name TCEB1), and Histone acetyltransferase p300 (EP300) (Figure 1) [21]. The protein ARNT is a representative HIF1β subunit, with which EPAS1 binds to form an active transcription factor HIF2. Similar role as a binding partner for a complete transcription factor HIF have also ARNT2 and ARNTL. Unlike the ubiquitous expression of ARNT, the expression of ARNT2 is restricted to early developmental stages and certain cell types in adult tissues, especially in the brain, neurons, and kidneys [45,46]. The protein ARNTL (also known as Brain and muscle ARNT-like 1, BMAL1) is best known to heterodimerize with protein CLOCK and regulates circadian rhythms. However, ARNTL was found to be a strong binding partner of EPAS1 in chondrocytes and heterodimers are potential regulators of the endochondral ossification process [47]. The key interactions responsible for the stability of EPAS1 are with prolyl hydroxylases EGLN (EGLN1, EGLN2, and EGLN3) and VHL. Heterodimer elongins ELOB and ELOC are a part of a multi-subunit VHL ubiquitin ligase complex [48]. The EP300 is a component of CBP/p300 co-activator for transcription of HIF target genes. Besides interactions deposited in STRING, the Reactome database includes additional six interactions: Mother against decapentaplegic homolog (SMAD) 3, fructose-1,6-bisphosphatase 1 (FBP1), Ubiquitin carboxyl-terminal hydrolase 8 and 20 (USP8 and USP20), RNA-binding protein 4 (RBM4), and Eukaryotic translation initiation factor 3 subunit E (EIF3E) [22]. Proteins SMAD3, FBP1, and EIF3E are repressors of EPAS1, by downregulating its transcriptional activity or inducing EPAS1 degradation [49,50,51]. Deubiquitinases USP8 and USP20 counteract VHL-mediated ubiquitylation and stabilize EPAS1 [52]. Protein RBM4 forms a transcription/translation activation complex with EPAS1 under hypoxia [30]. An extensive piece of research on EPAS1 interactions and other proteins in the HIF-EPO pathway and involvement in the molecular processes was recently published in two reviews by Tomc and Debeljak (2021) [51,53].

### 3.6. EPAS1 Downstream Targets

In the present study, we obtained data of 12 experimentally validated EPAS1 transcriptional targets (Table 3). For three target genes, *VEGFA*, *SLC2A1*, and *EPO*, their interaction with transcription factor EPAS1 is confirmed in more than one study (Table 3 and Figure 1). According to the enrichment analysis, those 12 genes were associated with 26 GO term biological processes: vascular associated smooth muscle cell development, cellular hyperosmotic response, mammary gland alveolus development, negative regulation of vascular permeability, response to hyperoxia, positive regulation of release of cytochrome c from mitochondria, positive regulation of epidermal growth factor receptor signaling pathway, cellular response to hypoxia, regulation of epithelial cell differentiation, response to glucocorticoid, positive regulation of angiogenesis, female pregnancy, positive regulation of peptidyl-tyrosine phosphorylation, positive regulation of epithelial cell proliferation, positive regulation of protein serine/threonine kinase activity, response to wounding, regulation of body fluid levels, response to nutrient levels, blood vessel morphogenesis, negative regulation of apoptotic process, response to bacterium, negative regulation of developmental process, response to oxygen-containing compound, intracellular signal transduction, immune system process, and negative regulation of metabolic process [24].

### 3.7. Association with Diseases

Dysregulation in *EPAS1* expression and its transcriptional activity has been associated with multiple diseases. Germline and somatic mutations were identified in several pathophysiological conditions including erythrocytosis, congenital heart disease, Lynch syndrome, and many types of cancer [59,60]. During tumor development and progression, hypoxia is a typical solid tumors microenvironment. As HIFs are critical transcription factors for adaptation to hypoxia, they can transactivate numerous genes involved in angiogenesis, anaerobic metabolism, apoptosis, cell migration, and cell cycle, which could promote cancerogenesis [32,61].

#### 3.7.1. Association with Cancers

**Figure 6:**
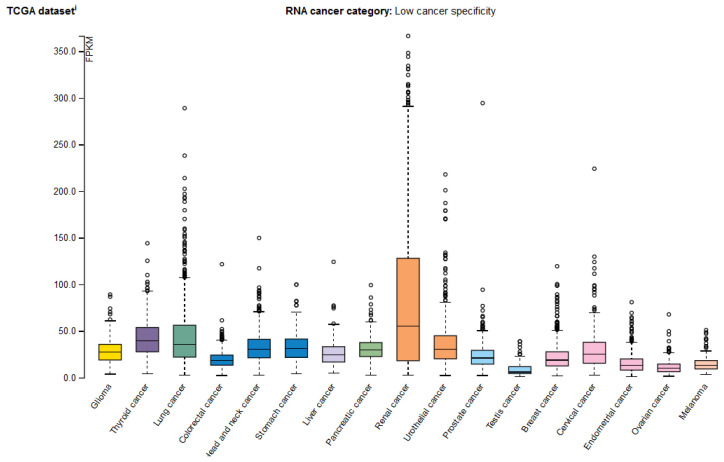
The *EPAS1* mRNA expression profiles in various cancer types from the Human Protein Atlas. The RNA-seq data is presented as a median number of fragments per kilobase of exon per million reads (FPKM) for 17 cancer types and is generated by The Cancer Genome Atlas (TCGA).

Several studies reported that the expression of *EPAS1* is associated with pathogenesis, progression, and prognosis of different cancer types, including renal cell carcinoma [62,63,64], non-small cell lung cancer (NSCLC) [65], hepatocellular carcinoma [66], neuroblastoma [67,68], glioma [69], colorectal carcinoma [35,70,71], esophageal squamous cell carcinoma [59], breast cancer [32,72], and paraganglioma and pheochromocytoma [73,74,75]. In the Human Protein Atlas, the *EPAS1* mRNA was detected in various cancer tissues (Figure 6) and EPAS1 is used as a prognostic marker in renal cancer [18]. 

**Figure 7:**
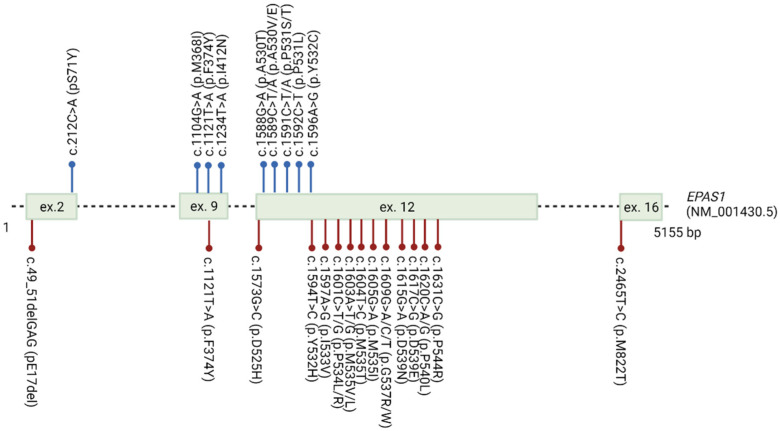
*EPAS1* variants detected in association with paraganglioma/pheochromocytoma and familial erythrocytosis ECYT4. Blue dots indicate somatic variants detected in patients with paraganglioma/pheochromocytoma. Red dots indicate germline variants detected in patients with ECYT4.

Somatic variants in the *EPAS1* nucleotide sequence were detected in various tumors. In the COSMIC database, 856 somatic variants are listed across the whole gene. The majority of variants are substitutions, while only 15 small insertions or deletions (INDELs) are reported [27]. Eleven somatic variants in exons 2, 9, and 12, with amino acid changes near the hydroxylation sites p.Pro405 and p.Pro531, have been extensively identified in the neuroendocrine tumors paraganglioma, pheochromocytoma, and somatostatinoma [73,74,75,76] (Figure 7).

#### 3.7.2. Association with Erythrocytosis

Based on the OMIM and Simple ClinVar databases, *EPAS1* is broadly associated with Familial erythrocytosis type 4 (ECYT4), a congenital disease with an excessive erythrocytes mass, elevated hemoglobin, and/or hematocrit. The review of the literature revealed 20 variants in the *EPAS1* gene associated with erythrocytosis; 19 missense substitutions, and one deletion in exons 2, 9, 12, and 16 [77] (Figure 7). However, in the database Global Variome shared LOVD, currently, only seven variants c.1601C>T, c.1603A>G, c.1604T>C, c.1609G>T, c.1609G>A, c.1617C>G, c.1620C>G are listed as pathogenic for ECYT4 [28].

## 4. Discussion

Since EPAS1 is involved in many molecular mechanisms for adaptation to hypoxia, the regulation of EPAS1 plays a critical role and is very complex. The precise mechanism of EPAS1 regulation reflects also in non-ubiquitous expression and distinct expression patterns over different tissues. The mechanisms of EPAS1 degradation at the protein level have been very well studied. Through literature, we gathered information of 16 different PTMs (Figure 1), including well-known hydroxylation, ubiquitination, acetylation, phosphorylation, methylation, and SUMOylation, with the majority of PTMs decreasing the stability of EPAS1 protein [13,43]. On the contrary, the research on the regulation of EPAS1 at the transcriptional level has been rather limited, but the list of additional mechanisms that regulate EPAS1 pathway is growing. In the study, we discussed several ways of transcriptional regulation from EPAS1 promoter-specific transcription factors [33,34] to chromatin modulation [31] and epigenetic changes [32] in the *EPAS1* promoter region. In addition, we found 14 non-coding regulatory RNAs (13 miRNAs and 1 lncRNA), which bind to the EPAS1 and potentially regulate *EPAS1* mRNA or protein levels [23,39]. However, to thoroughly understand the complex regulation of the *EPAS1* expression, many more present and future studies need to be reviewed.

Even though the expression of *EPAS1* throughout the body is more restricted than of its paralog HIF1α, it has an important role in the response to low body oxygenation and the development of various diseases. Over the years, it became evident that EPAS1 may play a broader role in tumorigenesis than previously thought. From the literature, we found that *EPAS1* expression is increased in nine different types of solid tumors, often associated with poor prognosis. However, this association is not absolute, as low EPAS1 protein expression was in some cases associated with advanced tumor stage [78]. In the Human protein atlas *EPAS1* mRNA was reported in 15 different cancer types, with the highest expression and as a prognostic marker in renal carcinoma (Figure 6). This is consistent with multiple pieces of evidence from the literature, which suggest a causal role of EPAS1 in renal cell carcinoma [62,63,79]. Due to the oncogenic effect of EPAS1 in cancerogenesis, EPAS1 is a potential therapeutic target for tumor suppression [59,80].

The obvious molecular mechanism for *EPAS1* induction in cancer is intratumoral hypoxia, but also genetic alterations. The highest number of studies associated somatic variants with paraganglioma and pheochromocytoma; however, variants were also found in other types of cancer, for instance in colon cancer [70] and NSCLC [65], but are not in the scope of this study. In almost all paraganglioma and pheochromocytoma cases, somatic variants were identified exclusively in the neuroendocrine tumor DNA, and absent from germline DNA. However, two studies identified inherited variant in the exon 9 (p.Phe374Tyr) [73,74]. In some cases, paraganglioma and pheochromocytoma patients also had erythrocytosis [73,75]. Findings of somatic *EPAS1* variants in separate and functionally distinct tumors from the same patient suggest that variants could occur in multiple cells during embryogenesis and predispose them to tumorigenesis [75]. The majority of somatic variants (7 out of 11) responsible for the paraganglioma/pheochromocytoma and the majority of germline variants (17 out of 20) responsible for the ECYT4 are located within the ODD domain, with amino acid changes at highly conserved residues in the vicinity to the primary hydroxylation point p.Pro531 (Figure 7). Surprisingly, almost all somatic variants in the exon 12 are located on the site of hydroxylated proline or towards N-terminus, while germline variants in the exon 12 associated with ECYT4 are located towards C-terminus from the proline. Functional studies in vitro confirmed that variants in the vicinity of the primary hydroxylation point impair hydroxylation by EGLN1 and subsequent binding of VHL protein, thus enhancing stability and activity of the EPAS1 protein [75,81,82,83,84,85].

With the literature review, we gathered 12 downstream targets (Table 3), but as EPAS1 is an important transcription factor, the number of transcriptional targets is much higher. To gain insight into a profound understanding of hypoxia-responsive mechanism, it is necessary to review a greater number of publications. Recently it became apparent that not only the expression of protein-coding genes is regulated by HIFs, but also the transcription of non-coding RNAs, such as miRNAs, lncRNAs, and antisense RNAs, which further play an important role in the development of aggressive tumors [86]. In this study we only focused on the transcriptional regulation of protein-coding genes by EPAS1. The majority of available studies regarding EPAS1 downstream transcriptional activity and its upstream regulation were performed on cancer cell lines and tissues, thus the enrolment of the regulatory mechanisms in healthy tissue must be carefully considered.

## 5. Conclusions

The purpose of the study was to combine the data of the *EPAS1* gene from different OMICs levels and visualize data as an integrative map. The *EPAS1* gene, located at chromosome 2, is transcribed into two protein-coding transcripts, which have enhanced expression in the lungs. Protein is located in the nucleus and cytosol and has eight domains important for EPAS1 activity and stability. At the regulomics level, we found: 31 upstream regulators, which bind to one of the two *EPAS1* promoters, overlapping one CpG island; 13 miRNAs that target the *EPAS1* mRNA and 1 lncRNA that targets the EPAS1 protein; and 16 PTMs that regulate proteasomal degradation of EPAS1 and its activity. According to the two databases of protein–protein interactions, the EPAS1 protein is directly interacting with 27 proteins. Also, we found interactions with 12 hypoxia-responsive target genes. Expression of *EPAS1* was detected in more than ten types of cancer and was also associated with other diseases, including erythrocytosis. Germline and somatic variants around important PTMs for EPAS1 stability were extensively associated with the development of the erythrocytosis type 4 and neuroendocrine tumors paraganglioma and pheochromocytoma.

With this study we presented a comprehensive view of the EPAS1 to facilitate a better understanding of the gene regulation, role, and activity and encourage supplementation of the data and further studies on EPAS1. We showed that the regulation of EPAS1 is a complex process on diverse OMICs levels. The number of publications on this topic is still rising and more studies need to be reviewed to gain profound knowledge into the EPAS1 regulation. There is a substantial research interest in the HIF family of genes, as they have been shown to be involved in cancerogenesis and are potential targets for cancer treatment. Therefore, we could expect more studies on EPAS1 in the future. To make the research more accessible, we propose fusion of diverse new data on EPAS1 in the form of integration maps, as presented in this study.

## Figures and Tables

**Table 1:** List of obtained *EPAS1*-specific promoter-associated proteins from different studies and cell lines.

Gene	Gene Entrez ID	Protein	Cell Line/Tissue	Reference
*IVNS1ABP*	10625	Influenza virus NS1A-binding protein	Neuroblastoma cell line SK-N-BE(2)c	[31]
*RAB11B*	9230	Ras-related protein Rab-11B		
*STOML2*	30968	Stomatin-like protein 2		
*PAFAH1B2*	5049	Platelet-activating factor acetylhydrolase 1b, subunit alpha2		
*HSPD1*	3329	60kDa heat shock protein 1		
*HSPA9*	3313	Stress-70 protein		
*PITPNB*	23760	Phosphatidylinositol transfer protein beta		
*ACTN1*	87	Alpha-actinin-1		
*HSPB1*	3315	Heat shock protein beta-1		
*PTS*	5805	6-pyruvoyl tetrahydrobiopterin synthase		
*SNRPB*	6628	Small nuclear ribonucleoprotein-associated proteins B and B’		
*NT5C2*	22978	Cytosolic purine 5′-nucleotidase,		
*U2AF1L4*	199746	Splicing factor U2AF 26 kDa subunit		
*MYH10*	4628	Myosin-10		
*HDX*	139324	Highly divergent homeobox		
*PSMA4*	5685	Proteasome subunit alpha type-4		
*HIST1H2AH*	85235	Histone H2A type 1-H		
*HIST4H4*	121504	Histone H4		
*HIST1H2BK*	85236	Histone H2B type 1-K		
*LMNB1*	4001	Lamin B1		
*CCAR1*	55749	Cell division cycle and apoptosis regulator 1		
*STK38*	11329	Serine/threonine protein kinase 38		
*ERH*	2079	Enhancer of rudimentary homolog		
*DLG1*	1739	Discs large homolog 1		
*SPIN1*	10927	Spindlin 1		
*EIF4B*	1975	Eukaryotic translation initiation factor 4B		
*NDFIP1*	80762	Nedd4 family interacting protein 1		
*MBD3*	53615	Methyl-CpG binding domain protein 3	Human breast cancer cell line MDA-MB-468	[32]
*TNFSF14/LIGHT* *NFKB2*	8740 4791	Tumor necrosis factor ligand superfamily member 14 Nuclear factor NF-kappa-B, p52 subunit	Human cancer cell lines HeLa and A549	[33]
*E2F1*	1869	Transcription factor E2F1	HeLa, 786-O, U2OS and HepG2	[34]

**Table 2:** The miRNAs confirmed to target human *EPAS1* with strong experimental evidence.

miRNA	Validation Methods	Cell Line/Tissue	Reference
hsa-miR-185-5p	Reporter assay, qPCR	Human umbilical vein endothelial cells (HUVEC)	[40]
hsa-miR-145-5p	Reporter assay, WB, qPCR	Neuroblastoma cell lines SH-SY5Y and SK-N-SH	[41]
hsa-miR-20a-5p	Reporter assay	Hepatic cellular carcinoma (tumor associated macrophages)	[42]
hsa-miR-17-5p	Reporter assay	Hepatic cellular carcinoma (tumor associated macrophages)	[42]

**Table 3:** Obtained downstream targets of the EPAS1 from the literature.

Target Gene		Species		Validation		Reference
Name (Synonim)	Entrez ID	Name	ID	Experimental Methods	Cell Line and/or Tissues	
*VEGFA (VEGF)*	7422	Human	9606	siRNA HIF1A/EPAS1	Renal carcinoma RCC4 and 786-O (no VHL function)	[54]
*VEGFA (VEGF)*	7422	Human	9606	DNA microarray and northern blot	Renal carcinoma 786-O (no HIF1A) and HEK293 TET-on cells	[55]
*VEGFA (VEGF)*	7422	Human	9606	siRNA HIF1A/EPAS1, ELISA	Renal carcinoma RCC4 and SKRC28	[56]
*SLC2A1 (GLUT1)*	6513	Human	9606	siRNA HIF1A/EPAS1	Renal carcinoma RCC4 and 786-O (no VHL function)	[54]
*SLC2A1 (GLUT1)*	6513	Human	9606	Overexpression of HIF1A/EPAS1, immunoblotting	Renal carcinoma 786-O, RCC4 and SKRC28	[56]
*PLAUR (uPAR)*	5329	Human	9606	siRNA HIF1A/EPAS1	Renal carcinoma RCC4 and 786-O (no VHL function)	[54]
*SERPINE1 (PAI-1)*	5054	Human	9606	siRNA HIF1A/EPAS1	Renal carcinoma RCC4 and 786-O (no VHL function)	[54]
*CCND1* (cyclin D1)	595	Human	9606	siRNA HIF1A/EPAS2, overexpression of HIF1A/EPAS1, immunoblotting	Renal carcinoma 786-O	[56]
*BNIP3*	664	Human	9606	Overexpression of HIF1A/EPAS1, immunoblotting	Renal carcinoma 786-O, RCC4 and SKRC28	[56]
*TGFA*	7039	Human	9606	siRNA HIF1A/EPAS1, ELISA	Renal carcinoma RCC4 and SKRC28	[56]
*PLIN2 (ADRP)*	123	Human	9606	DNA microarray and northern blot	Renal carcinoma 786-O (no HIF1A) and HEK293 TET-on cells	[55]
*NDRG1*	10397	Human	9606	DNA microarray and northern blot	Renal carcinoma 786-O (no HIF1A) and HEK293 TET-on cells	[55]
*ADM*	133	Human	9606	DNA microarray and northern blot	Renal carcinoma 786-O (no HIF1A) and HEK293 TET-on cells	[55]
*DNMT1*	1786	Human	9606	ChIP-qPCR, Luciferase assay	Lung cancer cell lines, HEK293 cells	[37]
*EPO*	3415	Mouse Human	10090 9606	Cre-mediated inactivation of HIF1A/EPAS1 in mice and siRNA in cell lines, RT-PCR, ChIP	Mice liver tissue and liver Hep3B cells	[57]
*EPO*	3415	Human	9606	siRNA HIF1A/EPAS1, immunoblotting, RNase protection assay	Liver Hep3B cells and neuroblastoma Kelly cells	[58]

## Data Availability

In the manuscript preparation, the following publicly available datasets were used: Ensembl, release 104 (https://www.ensembl.org/index.html) (accessed on 22 October 2021), National Centre for Biotechnology Information (NCBI) (https://www.ncbi.nlm.nih.gov/) (accessed on 22 October 2021), Human Protein Atlas, version 20.1 (https://www.proteinatlas.org/) (accessed on 22 October 2021), UniProt, release 2021_03 (https://www.uniprot.org/) (accessed on 9 November 2021), Methprimer (https://www.urogene.org/methprimer/) (accessed on 22 October 2021), STRING (https://string-db.org/) (accessed on 22 October 2021), Reactome (https://reactome.org/) (accessed on 22 October 2021), miRTarBase (https://mirtarbase.cuhk.edu.cn/) (accessed on 25 October 2021), Gene Ontology (GO) (http://geneontology.org/) (accessed on 22 October 2021), OMIM (https://omim.org/) (accessed on 22 October 2021), Simple ClinVar (http://simple-clinvar.broadinstitute.org/) (accessed on 22 October 2021), COSMIC (cancer.sanger.ac.uk) (accessed on 22 October 2021), LOVD (https://www.lovd.nl/) (accessed on 22 October 2021), UCSC Genome browser (https://genome.ucsc.edu/) (accessed on 22 October 2021).

## References

[1] Semenza G.L. (2014). Oxygen Sensing, Hypoxia-Inducible Factors, and Disease Pathophysiology. Annu. Rev. Pathol. Mech. Dis..

[2] Sutter C.H., Laughner E., Semenza G.L. (2000). Hypoxia-inducible factor 1alpha protein expression is controlled by oxygen-regulated ubiquitination that is disrupted by deletions and missense mutations. Proc. Natl. Acad. Sci. USA.

[3] Semenza G.L. (2012). Hypoxia-Inducible Factors in Physiology and Medicine. Cell.

[4] Semenza G.L., Wang G.L. (1992). A nuclear factor induced by hypoxia via de novo protein synthesis binds to the human erythropoietin gene enhancer at a site required for transcriptional activation. Mol. Cell. Biol..

[5] Ema M., Taya S., Yokotani N., Sogawa K., Matsuda Y., Fujii-Kuriyama Y. (1997). A novel bHLH-PAS factor with close sequence similarity to hypoxia-inducible factor 1alpha regulates the VEGF expression and is potentially involved in lung and vascular development. Proc. Natl. Acad. Sci. USA.

[6] Gu Y.-Z., Moran S.M., HogenEsch J.B., Wartman L., Bradfield C.A. (1998). Molecular Characterization and Chromosomal Localization of a Third alpha-Class Hypoxia Inducible Factor Subunit, HIF3alpha. Gene Expr..

[7] Hogenesch J.B., Chan W.K., Jackiw V.H., Brown R.C., Gu Y.-Z., Pray-Grant M., Perdew G.H., Bradfield C.A. (1997). Characterization of a Subset of the Basic-Helix-Loop-Helix-PAS Superfamily That Interacts with Components of the Dioxin Signaling Pathway. J. Biol. Chem..

[8] Lee F.S., Percy M.J. (2011). The HIF Pathway and Erythrocytosis. Annu. Rev. Pathol. Mech. Dis..

[9] Semenza G.L. (2009). Involvement of oxygen-sensing pathways in physiologic and pathologic erythropoiesis. Blood.

[10] Mole D.R., Blancher C., Copley R.R., Pollard P., Gleadle J., Ragoussis J., Ratcliffe P. (2009). Genome-wide Association of Hypoxia-inducible Factor (HIF)-1alpha and HIF-2alpha DNA Binding with Expression Profiling of Hypoxia-inducible Transcripts. J. Biol. Chem..

[11] Wenger R.H., Stiehl D.P., Camenisch G. (2005). Integration of Oxygen Signaling at the Consensus HRE. Sci. STKE.

[12] Slemc L., Kunej T. (2016). Transcription factor HIF1A: Downstream targets, associated pathways, polymorphic hypoxia response element (HRE) sites, and initiative for standardization of reporting in scientific literature. Tumor Biol..

[13] Dengler V.L., Galbraith M., Espinosa J.M. (2013). Transcriptional regulation by hypoxia inducible factors. Crit. Rev. Biochem. Mol. Biol..

[14] Majmundar A.J., Wong W.J., Simon M.C. (2010). Hypoxia-Inducible Factors and the Response to Hypoxic Stress. Mol. Cell.

[15] Kunej T. (2021). Integrative Map of *HIF1A* Regulatory Elements and Variations. Genes.

[16] Yates A.D., Achuthan P., Akanni W., Allen J., Alvarez-Jarreta J., Amode M.R., Armean I., Azov A., Bennett E.R., Bhai J. (2019). Ensembl 2020. Nucleic Acids Res..

[17] Coordinators N.R., Agarwala R., Barrett T., Beck J., Benson D.A., Bollin C., Bolton E., Bourexis D., Brister J.R., Bryant S.H. (2017). Database resources of the National Center for Biotechnology Information. Nucleic Acids Res..

[18] Uhlén M., Fagerberg L., Hallström B.M., Lindskog C., Oksvold P., Mardinoglu A., Sivertsson A., Kampf C., Sjöstedt E., Asplund A. (2015). Proteomics. Tissue-based map of the human proteome. Science.

[19] (2018). The UniProt Consortium UniProt: A worldwide hub of protein knowledge. Nucleic Acids Res..

[20] Li L.-C., Dahiya R. (2002). MethPrimer: Designing primers for methylation PCRs. Bioinformatics.

[21] Szklarczyk D., Gable A.L., Lyon D., Junge A., Wyder S., Huerta-Cepas J., Simonovic M., Doncheva N.T., Morris J.H., Bork P. (2018). STRING v11: Protein–protein association networks with increased coverage, supporting functional discovery in genome-wide experimental datasets. Nucleic Acids Res..

[22] Jassal B., Matthews L., Viteri G., Gong C., Lorente P., Fabregat A., Sidiropoulos K., Cook J., Gillespie M., Haw R. (2019). The reactome pathway knowledgebase. Nucleic Acids Res..

[23] Huang H.-Y., Lin Y.-C., Li J., Huang K.-Y., Shrestha S., Hong H.-C., Tang Y., Chen Y.-G., Jin C.-N., Yu Y. (2019). miRTarBase 2020: Updates to the experimentally validated microRNA–target interaction database. Nucleic Acids Res..

[24] Mi H., Muruganujan A., Ebert D., Huang X., Thomas P.D. (2018). PANTHER version 14: More genomes, a new PANTHER GO-slim and improvements in enrichment analysis tools. Nucleic Acids Res..

[25] Amberger J.S., Bocchini C.A., Scott A.F., Hamosh A. (2018). OMIM.org: Leveraging knowledge across phenotype–gene relationships. Nucleic Acids Res..

[26] Pérez-Palma E., Gramm M., Nürnberg P., May P., Lal D. (2019). Simple ClinVar: An interactive web server to explore and retrieve gene and disease variants aggregated in ClinVar database. Nucleic Acids Res..

[27] Tate J.G., Bamford S., Jubb H.C., Sondka Z., Beare D.M., Bindal N., Boutselakis H., Cole C.G., Creatore C., Dawson E. (2018). COSMIC: The Catalogue of Somatic Mutations in Cancer. Nucleic Acids Res..

[28] Fokkema I., Taschner P.E.M., Schaafsma G., Celli J., Laros J.F., Dunnen J.T.D. (2011). LOVD v.2.0: The next generation in gene variant databases. Hum. Mutat..

[29] Pirih N., Kunej T. (2018). An Updated Taxonomy and a Graphical Summary Tool for Optimal Classification and Comprehension of Omics Research. OMICS.

[30] Uniacke J., Holterman C., Lachance G., Franovic A., Jacob M.D., Fabian M.R., Payette J., Holcik M., Pause A., Lee S. (2012). An oxygen-regulated switch in the protein synthesis machinery. Nature.

[31] Hamidian A., Vaapil M., von Stedingk K., Fujita T., Persson C., Eriksson P., Veerla S., De Preter K., Speleman F., Fujii H. (2018). Promoter-associated proteins of EPAS1 identified by enChIP-MS—A putative role of HDX as a negative regulator. Biochem. Biophys. Res. Commun..

[32] Cui J., Duan B., Zhao X., Chen Y., Sun S., Deng W., Zhang Y., Du J., Chen Y., Gu L. (2016). MBD3 mediates epigenetic regulation on EPAS1 promoter in cancer. Tumor Biol..

[33] D’Ignazio L., Batie M., Rocha S. (2018). TNFSF14/LIGHT, a Non-Canonical NF-kappaB Stimulus, Induces the HIF Pathway. Cells.

[34] Moniz S., Bandarra D., Biddlestone J., Campbell K., Komander D., Bremm A., Rocha S. (2015). Cezanne regulates E2F1-dependent HIF2alpha expression. J. Cell Sci..

[35] Rawłuszko-Wieczorek A.A., Horbacka K., Krokowicz P., Misztal M., Jagodzinski P. (2014). Prognostic Potential of DNA Methylation and Transcript Levels of HIF1A and EPAS1 in Colorectal Cancer. Mol. Cancer Res..

[36] Pan R., Zhou C., Dai J., Ying X., Yu H., Zhong J., Zhang Y., Wu B., Mao Y., Wu D. (2018). Endothelial PAS domain protein 1 gene hypomethylation is associated with colorectal cancer in Han Chinese. Exp. Ther. Med..

[37] Xu X., Bao Y., Wang X., Yan F., Guo S., Ma Y., Xu D., Jin L., Xu J., Wang J. (2018). Hypoxic-stabilized EPAS1 proteins transactivate DNMT1 and cause promoter hypermethylation and transcription inhibition of EPAS1 in non-small cell lung cancer. FASEB J..

[38] Yoo S., Takikawa S., Geraghty P., Argmann C., Campbell J., Lin L., Huang T., Tu Z., Feronjy R., Spira A. (2015). Integrative Analysis of DNA Methylation and Gene Expression Data Identifies EPAS1 as a Key Regulator of COPD. PLoS Genet..

[39] Zhu T., Wang Z., Wang G., Hu Z., Ding H., Li R., Sun J. (2020). Long non-coding RNA ZFAS1 promotes the expression of EPAS1 in gastric cardia adenocarcinoma. J. Adv. Res..

[40] Ho J.J.D., Metcalf J.L., Yan M.S., Turgeon P.J., Wang J.J., Chalsev M., Petruzziello-Pellegrini T.N., Tsui A.K.Y., He J.Z., Dhamko H. (2012). Functional Importance of Dicer Protein in the Adaptive Cellular Response to Hypoxia. J. Biol. Chem..

[41] Zhang H., Pu J., Qi T., Qi M., Yang C., Li S., Huang K., Zheng L., Tong Q. (2012). MicroRNA-145 inhibits the growth, invasion, metastasis and angiogenesis of neuroblastoma cells through targeting hypoxia-inducible factor 2 alpha. Oncogene.

[42] Xu Z., Zhao L., Zhu L.-Y., He M., Zheng L., Wu Y. (2013). MicroRNA-17, 20a Regulates the Proangiogenic Function of Tumor-Associated Macrophages via Targeting Hypoxia-Inducible Factor 2alpha. PLoS ONE.

[43] Albanese A., Daly L., Mennerich D., Kietzmann T., Sée V. (2020). The Role of Hypoxia-Inducible Factor Post-Translational Modifications in Regulating Its Localisation, Stability, and Activity. Int. J. Mol. Sci..

[44] Dioum E.M., Chen R., Alexander M.S., Zhang Q., Hogg R.T., Gerard R.D., Garcia J.A. (2009). Regulation of Hypoxia-Inducible Factor 2alpha Signaling by the Stress-Responsive Deacetylase Sirtuin 1. Science.

[45] Rankin E.B., Giaccia A.J. (2008). The role of hypoxia-inducible factors in tumorigenesis. Cell Death Differ..

[46] Freeburg P.B., Abrahamson D.R. (2004). Divergent Expression Patterns for Hypoxia-Inducible Factor-1beta and Aryl Hydrocarbon Receptor Nuclear Transporter-2 in Developing Kidney. J. Am. Soc. Nephrol..

[47] Saito T., Kawaguchi H. (2010). HIF-2alpha as a possible therapeutic target of osteoarthritis. Osteoarthr. Cartil..

[48] Kaelin W.G. (2017). The VHL Tumor Suppressor Gene: Insights into Oxygen Sensing and Cancer. Trans. Am. Clin. Clim. Assoc..

[49] Ma X., Das N.K., Castillo C., Gourani A., Perekatt A.O., Verzi M.P., Shah Y.M. (2019). SMAD family member 3 (SMAD3) and SMAD4 repress HIF2alpha-dependent iron-regulatory genes. J. Biol. Chem..

[50] Ning X.-H., Li T., Gong Y.-Q., He Q., Shen Q., Peng S.-H., Wang J.-Y., Chen J.-C., Guo Y.-L., Gong K. (2016). Association between FBP1 and hypoxia-related gene expression in clear cell renal cell carcinoma. Oncol. Lett..

[51] Tomc J., Debeljak N. (2021). Molecular Insights into the Oxygen-Sensing Pathway and Erythropoietin Expression Regulation in Erythropoiesis. Int. J. Mol. Sci..

[52] Kubaichuk K., Kietzmann T. (2019). Involvement of E3 Ligases and Deubiquitinases in the Control of HIF-alpha Subunit Abundance. Cells.

[53] Tomc J., Debeljak N. (2021). Molecular Pathways Involved in the Development of Congenital Erythrocytosis. Genes.

[54] Carroll V., Ashcroft M. (2006). Role of Hypoxia-Inducible Factor (HIF)-1alpha versus HIF-2alpha in the Regulation of HIF Target Genes in Response to Hypoxia, Insulin-Like Growth Factor-I, or Loss of von Hippel-Lindau Function: Implications for Targeting the HIF Pathway. Cancer Res..

[55] Hu C.-J., Wang L.-Y., Chodosh L.A., Keith B., Simon M.C. (2003). Differential Roles of Hypoxia-Inducible Factor 1alpha (HIF-1alpha) and HIF-2alpha in Hypoxic Gene Regulation. Mol. Cell. Biol..

[56] Raval R.R., Lau K.W., Tran M.G.B., Sowter H.M., Mandriota S.J., Li J.-L., Pugh C., Maxwell P., Harris A.L., Ratcliffe P.J. (2005). Contrasting Properties of Hypoxia-Inducible Factor 1 (HIF-1) and HIF-2 in von Hippel-Lindau-Associated Renal Cell Carcinoma. Mol. Cell. Biol..

[57] Rankin E., Biju M.P., Liu Q., Unger T.L., Rha J., Johnson R., Simon M.C., Keith B., Haase V.H. (2007). Hypoxia-inducible factor–2 (HIF-2) regulates hepatic erythropoietin in vivo. J. Clin. Investig..

[58] Warnecke C., Zaborowska Z., Kurreck J., Erdmann V.A., Frei U., Wiesener M., Eckardt K. (2004). Differentiating the functional role of hypoxia-inducible factor (HIF)-1alpha and HIF-2alpha (EPAS-1) by the use of RNA interference: Erythropoietin is a HIF-2alpha target gene in Hep3B and Kelly cells. FASEB J..

[59] Islam F., Gopalan V., Law S., Lam A.K., Pillai S. (2020). Molecular Deregulation of EPAS1 in the Pathogenesis of Esophageal Squamous Cell Carcinoma. Front. Oncol..

[60] Gaspersic J., Kristan A., Kunej T., Zupan I.P., Debeljak N. (2020). Erythrocytosis: Genes and pathways involved in disease development. Blood Transfus..

[61] Jun J.C., Rathore A., Younas H., Gilkes D., Polotsky V.Y. (2017). Hypoxia-Inducible Factors and Cancer. Curr. Sleep Med. Rep..

[62] Cho H., Du X., Rizzi J.P., Liberzon E., Chakraborty A.A., Gao W., Carvo I., Signoretti S., Bruick R.K., Josey J.A. (2016). On-target efficacy of a HIF-2alpha antagonist in preclinical kidney cancer models. Nature.

[63] Meléndez-Rodríguez F., Roche O., Sanchez-Prieto R., Aragonés J. (2018). Hypoxia-Inducible Factor 2-Dependent Pathways Driving Von Hippel–Lindau-Deficient Renal Cancer. Front. Oncol..

[64] Han S.S., Yeager M., Moore L.E., Wei M.-H., Pfeiffer R., Toure O., Purdue M.P., Johansson M., Scelo G., Chung C.C. (2011). The chromosome 2p21 region harbors a complex genetic architecture for association with risk for renal cell carcinoma. Hum. Mol. Genet..

[65] Putra A.C., Eguchi H., Lee K.L., Yamane Y., Gustine E., Isobe T., Nishiyama M., Hiyama K., Poellinger L., Tanimoto K. (2015). The A Allele at rs13419896 of EPAS1 Is Associated with Enhanced Expression and Poor Prognosis for Non-Small Cell Lung Cancer. PLoS ONE.

[66] Bangoura G., Liu Z.S., Qian Q., Jiang C.Q., Yang G.F., Jing S. (2007). Prognostic significance of HIF-2alpha/EPAS1 expression in hepatocellular carcinoma. World J. Gastroenterol..

[67] Mengelbier L.H., Fredlund E., Löfstedt T., Noguera R., Navarro S., Nilsson H., Pietras A., Vallon-Christersson J., Borg A., Gradin K. (2006). Recruitment of HIF-1alpha and HIF-2alpha to common target genes is differentially regulated in neuroblastoma: HIF-2alpha promotes an aggressive phenotype. Cancer Cell.

[68] Påhlman S., Mohlin S. (2017). Hypoxia and hypoxia-inducible factors in neuroblastoma. Cell Tissue Res..

[69] Li Z., Bao S., Wu Q., Wang H., Eyler C., Sathornsumetee S., Shi Q., Cao Y., Lathia J., McLendon R.E. (2009). Hypoxia-Inducible Factors Regulate Tumorigenic Capacity of Glioma Stem Cells. Cancer Cell.

[70] Islam F., Gopalan V., Lu C.T., Pillai S., Lam A.K. (2021). Identification of novel mutations and functional impacts of EPAS1 in colorectal cancer. Cancer Med..

[71] Yoshimura H., Dhar D.K., Kohno H., Kubota H., Fujii T., Ueda S., Kinugasa S., Tachibana M., Nagasue N. (2004). Prognostic Impact of Hypoxia-Inducible Factors 1alpha and 2alpha in Colorectal Cancer Patients: Correlation with Tumor Angiogenesis and Cyclooxygenase-2 Expression. Clin. Cancer Res..

[72] Helczynska K., Larsson A.-M., Mengelbier L.H., Bridges E., Fredlund E., Borgquist S., Landberg G., Påhlman S., Jirström K. (2008). Hypoxia-Inducible Factor-2alpha Correlates to Distant Recurrence and Poor Outcome in Invasive Breast Cancer. Cancer Res..

[73] Lorenzo F.R., Yang C., Fui M.N.T., Vankayalapati H., Zhuang Z., Huynh T., Grossmann M., Pacak K., Prchal J.T. (2012). A novel EPAS1/HIF2A germline mutation in a congenital polycythemia with paraganglioma. J. Mol. Med..

[74] Welander J., Andreasson A., Brauckhoff M., Bäckdahl M., Larsson C., Gimm O., Söderkvist P. (2014). Frequent EPAS1/HIF2alpha exons 9 and 12 mutations in non-familial pheochromocytoma. Endocrine-Related Cancer.

[75] Zhuang Z., Yang C., Lorenzo F., Merino M., Fojo T., Kebebew E., Popovic V., Stratakis C.A., Prchal J.T., Pacak K. (2012). Somatic HIF2A Gain-of-Function Mutations in Paraganglioma with Polycythemia. N. Engl. J. Med..

[76] Toledo R.A., Qin Y., Srikantan S., Morales N.P., Li Q., Deng Y., Kim S.-W., Pereira M.A., Toledo S.P., Su X. (2013). In vivo and in vitro oncogenic effects of HIF2A mutations in pheochromocytomas and paragangliomas. Endocrine-Related Cancer.

[77] Kristan A., Debeljak N., Kunej T. (2019). Genetic variability of hypoxia-inducible factor alpha (HIFA) genes in familial erythrocytosis: Analysis of the literature and genome databases. Eur. J. Haematol..

[78] Semenza G.L. (2009). Defining the role of hypoxia-inducible factor 1 in cancer biology and therapeutics. Oncogene.

[79] Kaelin W.G. (2009). Treatment of kidney cancer: Insights provided by the VHL tumor-suppressor protein. Cancer.

[80] Li T., Mao C., Wang X., Shi Y., Tao Y. (2020). Epigenetic crosstalk between hypoxia and tumor driven by HIF regulation. J. Exp. Clin. Cancer Res..

[81] Furlow P.W., Percy M.J., Sutherland S., Bierl C., McMullin M.F., Master S.R., Lappin T.R.J., Lee F.S. (2009). Erythrocytosis-associated HIF-2alpha Mutations Demonstrate a Critical Role for Residues C-terminal to the Hydroxylacceptor Proline. J. Biol. Chem..

[82] Gale D., Harten S.K., Reid C.D.L., Tuddenham E.G.D., Maxwell P.H. (2008). Autosomal dominant erythrocytosis and pulmonary arterial hypertension associated with an activating HIF2 alpha mutation. Blood.

[83] Percy M.J., Beer P.A., Campbell G., Dekker A.W., Green A.R., Oscier D., Rainey M.G., Van Wijk R., Wood M., Lappin T.R.J. (2008). Novel exon 12 mutations in the HIF2A gene associated with erythrocytosis. Blood.

[84] Percy M.J., Chung Y.J., Harrison C., Mercieca J., Hoffbrand A.V., Dinardo C.L., Santos P.C., Fonseca G.H., Gualandro S.F., Pereira A.C. (2012). Two new mutations in the HIF2A gene associated with erythrocytosis. Am. J. Hematol..

[85] Perrotta S., Stiehl D.P., Punzo F., Scianguetta S., Borriello A., Bencivenga D., Casale M., Nobili B., Fasoli S., Balduzzi A. (2013). Congenital erythrocytosis associated with gain-of-function HIF2A gene mutations and erythropoietin levels in the normal range. Haematologica.

[86] Choudhry H., Mole D.R. (2015). Hypoxic regulation of the noncoding genome and NEAT1. Briefings Funct. Genom..

